# Spreading of *Pasteurella multocida* Infection in a Pet Rabbit Breeding and Possible Implications on Healed Bunnies

**DOI:** 10.3390/vetsci9060301

**Published:** 2022-06-18

**Authors:** Francesco D’Amico, Gaia Casalino, Giancarlo Bozzo, Antonio Camarda, Roberto Lombardi, Michela Maria Dimuccio, Elena Circella

**Affiliations:** Department of Veterinary Medicine, University of Bari “Aldo Moro”, S. P. Casamassima km 3, 70010 Valenzano, Italy; francesco.damico@uniba.it (F.D.); gaia.casalino@uniba.it (G.C.); giancarlo.bozzo@uniba.it (G.B.); antonio.camarda@uniba.it (A.C.); roberto.lombardi@uniba.it (R.L.); michela.dimuccio@uniba.it (M.M.D.)

**Keywords:** pet rabbit, *Pasteurella multocida*, virulence genes, vaccine

## Abstract

The number of pets such as dogs, cats, rabbits, and parrots has increased in European families. Social benefits to owners such as decreasing feelings of loneliness and anxiety are provided by pets which are also used in Animal-assisted Therapy (AAT). Nevertheless, human-animal interactions are also associated with health problems including allergies, asthma, and zoonosis. Rabbits may carry potential pathogens for humans. One of the most common bacteria that colonizes the oro-pharynx and the upper respiratory tract of rabbits is *Pasteurella (P.) multocida*. Transmission of the infection to humans results from scratches, licks, and bites but it also can occur from the inhalation of air particles containing the microorganism. Immunocompromised people or persons with pulmonary disorders are particularly susceptible to the infection. Infected rabbits may carry *P. multocida* with or without clinical signs. In this paper, the sensitivity to antibiotics and the invasiveness ability of *P. multocida* identified in a farm of pet rabbits affected by severe pasteurellosis were investigated. The strain was *P. multocida* belonging to capsular type A which is the type most often detected in humans. The identified strain was susceptible to the tested antibiotics, but it appeared equipped with several virulence genes which are responsible for fimbriae production, adhesion processes to host cells, enzyme production, and are involved in iron acquisition processes. These findings are of particular interest because rabbits recovered from pasteurellosis very often become carriers of the bacteria. Therefore, we suggest considering *P. multocida* screening in the routine medical checks of rabbits, especially if they are meant to be companion animals for children and elder people, given that the transmission of the pathogen cannot be excluded.

## 1. Introduction

In recent years, the presence of pets in European families has increased and their number nowadays exceeds 300 million [1]. After dogs and cats, unconventional animals such as small mammals (30 million) and pet birds (52 million) represent a significant part of the total number of pets [1]. In particular, rabbits are increasingly common in the home environment in some countries such as the United Kingdom [2]. Pets provide great social benefits to owners such as decreasing feelings of loneliness and anxiety and increasing opportunities for socialization [3]. Moreover, dogs, cats, horses, rabbits, and hamsters are frequently used in Animal-assisted Therapy (AAT). Despite these benefits, human-animal interactions are associated with risks of health problems, including allergies, asthma, animal bites, and scratches [4]. In addition, pets are not always able to provide a positive effect in case of mental illness or untreated stress in people [3]. Companion animals can also transmit potential pathogens to humans. Zoonotic agents can be transmitted both through direct contact with infected animals and indirectly through environmental contamination [2]. People aged over 65 years and children younger than 5 years are the most susceptible to such infections. Moreover, immunocompromised persons and pregnant women are particularly exposed to the risk of pet-induced zoonoses [5].

Psittacosis is the most known zoonosis transmitted by pet birds. *Chlamydia (C.) psittaci* can be present in 40% of pet birds, most prevalently parrots but also other species such as canary [6,7,8]. Infected birds spread *C. psittaci* by excreta, nasal, and ocular secretions. The transmission of *C. psittaci* to humans occurs through the inhalation of the pathogen spread in the air and dust contaminated by respiratory secretions and feces from infected birds. In addition, transmission via bird bites and transmission beak to mouth have been reported [9]. In humans, *C. psittaci* can cause asymptomatic infections or acute infections characterized by flu-like illness, mild to severe atypical pneumonia, and systemic disease, which can be fatal in untreated patients [10]. Pet birds may carry also other pathogens such as nontyphoidal *Salmonella* [11], *Campylobacter jejuni* [12], and *Mycobacterium avium* subs. *avium* [13] to humans.

Zoonoses are also associated with rabbits. Dermatophytosis in rabbits is caused by *Trichophyton mentagrophytes*, *Microsporum* (*M.*) *canis*, and *M. gypseum* which represent the most common agents of zoonosis in rabbits [2,14]. Rabbits can also carry several parasites such as *Encephalitozoon cuniculi* [15], *Cryptosporidium* spp. [16], *Giardia* spp. [17], and *Tricostrongylus* spp. [18] which can induce zoonoses.

Rabbits may also host viruses and bacteria which can be potential pathogens for humans. The hepatitis E virus has been recently identified in rabbits [19]. In France, a recent study reported evidence of natural SARS-CoV-2 infection in rabbits that was attributed to direct contact with human patients [20]. A recent study carried out in experimental conditions highlighted the evidence of viral shedding in infected rabbits, despite the low seroprevalence and the lack of clinical signs [21].

The detection of *Mycobacterium avium* subsp. *hominissuis* has been reported in domestic rabbits [22]. Moreover, three different *Bartonella* (*B.*) species, *B. vinsonii* subsp. *berkhoffii*, *B. alsatica*, and *B. rochalimae,* were identified in fleas from rabbits, although they were wild animals [23]. One of the most common bacteria of rabbits is *Pasteurella (P.) multocida* which colonizes the upper respiratory tract and the oro-pharynx. *P. multocida* is a small coccobacillus, pleomorphic, Gram-negative, facultative aerobic-anaerobic, asporigenous, immobile bacterium with a fermentative metabolism, belonging to the *Pasteurellaceae* family. *P. multocida* is characterized by considerable genetic and antigenic variability. The strains are classified into 5 capsular types or serogroups (A, B, D, E, and F), based on the antigenic characteristics of the capsule polysaccharides, which are further classified into 16 somatic serotypes, based on lipopolysaccharide antigens, using serological techniques. Infected rabbits may carry *P. multocida* without showing clinical signs or they may be affected by heavy clinical syndromes. Although the symptoms frequently develop when immunosuppression, stress, or adverse environmental conditions occur, the pathogenicity of *P. multocida* is influenced by the presence of several virulence factors (VFs) prevalently identified in capsule proteins and lipopolysaccharides [24,25]. Many other virulence genes that may be involved in the pathogenicity of *P. multocida* include fimbriae, adhesion, and colonization factors (*ptfa*, *fimA*, *pfhA, and tadD*), iron regulation factors and protein acquisition (*exbB*, *exbD*, *tonB*, *hgbA*, *hgbB*, *tbpA*, and *fur*), superoxide dismutase (*sodA* and *sodC*), dermonecrotic toxins (*toxA*), a variety of Outer Membrane Proteins (*OMPs*) as protective factors (*ompA*, *ompH*, *omp87*, and *plpB*), and neuraminidase (*nanB* and *nanH*) [25,26,27,28,29]. Therefore, the variability of the microorganism is also evident in the multiple clinical forms associated with pasteurellosis in rabbits, which mainly include respiratory tract diseases but also otitis, pyometra, mastitis, orchitis, abortions, subcutaneous abscesses, and acute septicemic forms [30]. Transmission of *P. multocida* to humans is usually due to bites, scratches, and licks by companion animals with the development of local inflammation and occasional abscess formation and, sometimes, ascending infection [5,9,31,32]. Wound infections may result in several other clinical forms such as lymphangitis, joint infections, cellulitis, pneumonia, endocarditis, meningitis, urinary infections, and sepsis [33,34]. Immunocompromised people or persons with pulmonary disorders are particularly susceptible to systemic infection [35]. Additionally, the transmission can occur following the inhalation of air particles containing the microorganism [31,36]. Therefore, *P. multocida* infections in humans are associated with respiratory symptoms, such as pneumonia, tracheobronchitis, lung abscesses, and pulmonary emphysema [37].

The significant increase in the number of families who choose a rabbit as a companion animal has led to giving more attention to *Pasteurella multocida* infection in rabbits as well. The aim of this work is to achieve more information on *P. multocida* from pet rabbits starting from a severe outbreak of pasteurellosis that occurred on a farm of pet rabbits routinely sold as companion animals and to discuss a preventive approach in order to reduce the spread of *P. multocida* among the animals.

## 2. Materials and Methods

### 2.1. History

The flock of pet rabbits was evaluated because they were suffering from respiratory syndrome cyclically for about two years. The flock was in Corato, BA, southern Italy, and consisted of about 100 rabbits, 90 females and 10 males, all reared to breed. The animals were Lion’s head and lop rabbits. They were housed in a shed where a climate control system ensured an ambient temperature of 15–18 °C and relative humidity of approximately 65%. The rabbits were fed with pellets, fresh vegetables, and hay. Each rabbit was housed in a single cage equipped with a drinking bottle and a feeder. Cages and sheds were regularly cleaned. The reproductive activity in the flock was scheduled throughout the year except for July and August, for the lacking demand for bunnies by customers in that period. The female animals were usually introduced in the cages of male rabbits and left for three days to allow natural mating and pregnancy. The rate of fertility was about 95%. The bunnies were regularly sold to private individuals and pet shops.

### 2.2. Clinical Findings

**Figure 1:**
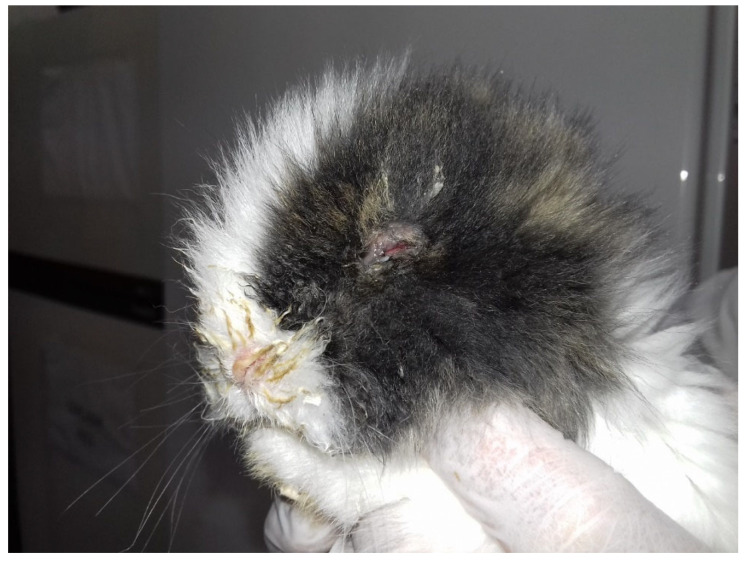
Left side of a bunny with severe clinical signs. Note the swollen eyelids and the purulent discharge on the hair around the nostrils.

The respiratory syndrome occurred in both bunnies and adults. Conjunctivitis was the most evident clinical sign. This symptom was particularly observed in bunnies (Figure 1), and it usually appeared at 13–14 days of age, after a few days since the opening of the eyes.

**Figure 2:**
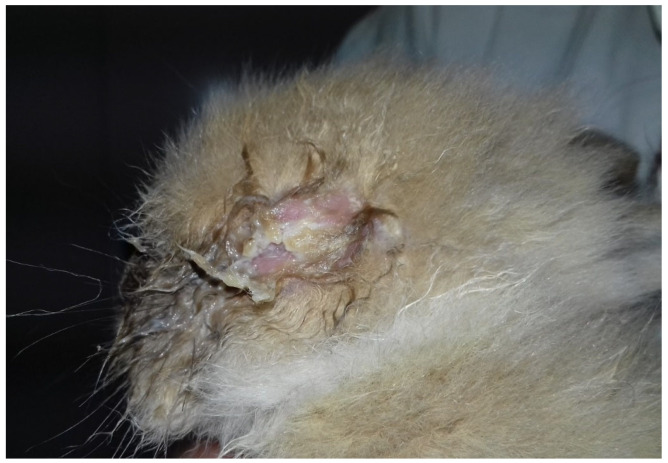
Left side of the head of a bunny with severe conjunctivitis. Note the purulent ocular discharge that occludes the eyelid.

Conjunctivitis started with catarrhal discharge, but the ocular discharge quickly became muco-purulent and occluded the eyelids (Figure 2).

**Figure 3:**
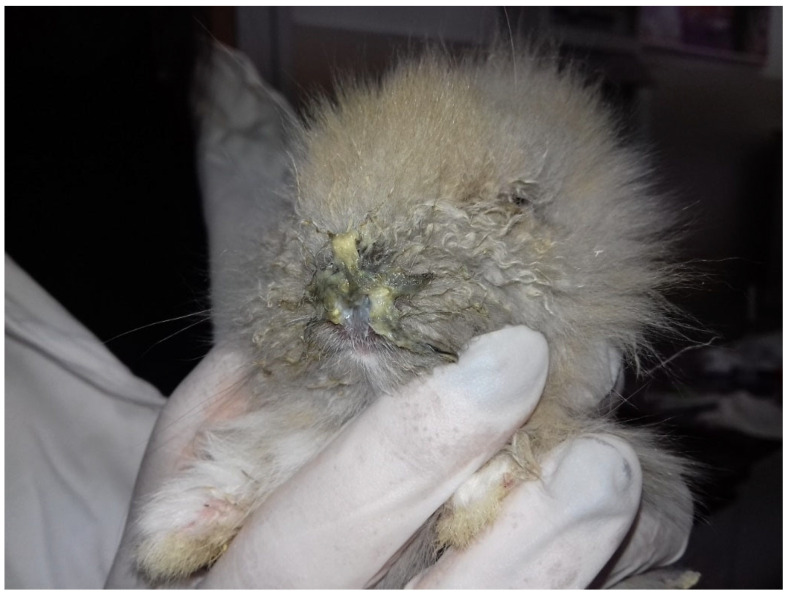
Bunny with severe purulent rhinitis.

Purulent rhinitis was frequently observed (Figure 3) and was often associated with the ocular lesion.

Affected bunnies were often undersized. The number of bunnies involved in the syndrome in each litter was variable. In fact, all the litter showed clinical signs in some cases, while only some bunnies were symptomatic in others. Sometimes, all the litter appeared healthy, but respiratory syndrome often appeared later, at the weaning time or after the sale of the bunnies. Breeders showed chronic rhinitis with muco-purulent discharge, but mothers of affected litters were not always symptomatic. Subcutaneous abscesses and torticollis were rarely observed only in adults. Moreover, some cases of late abortus were reported in lop rabbits. The animals were treated locally with ophthalmic ointments (chloramphenicol and chlortetracycline) by the owner which, cyclically, also administered trimethoprim/sulfamethoxazole via drinking water without improvements in the clinical conditions. Nasal and conjunctival swabs were collected from twelve symptomatic rabbits for laboratory investigations.

### 2.3. Bacteriology

Nasal and conjunctival swabs were plated on blood agar (Triptic Soy agar, Basingstoke, UK, supplemented with 5% sheep blood) and incubated at 37 °C for 24 h. Blood agar allows the growth of several different bacteria which can be associated with respiratory disorders (such as *Pasteurella multocida*, *Bordetella bronchiseptica*, and *Staphylococcus aureus)* or not associated with (*Micrococcus* spp., *Staphylococcus* spp., *Streptococcus* spp., and *Pseudomonas* spp.).

Three to five colonies from each plate were tested by Multiplex PCR according to [38] with some modifications to obtain the identification as *Pasteurella multocida* (*P. multocida*) and contextually to define the capsular type. Briefly, DNA extraction was carried out by boiling for 10 min. The primer set used in the PCR reaction is reported in Table 1. The PCR mixture consisted of 12.5 µL of 1X Platinum Mastermix (Thermo Scientific, Milan, Italy), containing 0.2 µL and 0.3 µL (50 pmol/µL primary concentration) of primer pair for specie identification and primer pairs for capsular types, respectively, and ultra-pure nuclease-free water (Thermo Scientific) until a final volume of 25 µL. Cycling conditions were as follows: 95 °C for 5 min, 35 cycles, each with 95 °C for 30 s, 55 °C for 30 s, 72 °C for 1 min and 10 s, and a final extension at 72 °C for 10 min. The PCR products were loaded for electrophoresis using a 1.5% agarose gel, which was stained with ethidium bromide. The reaction was visualized using the Gel Doc-It image analyzer (UVP, Upland, CA, USA).

### 2.4. Pathogenicity Genes Investigation

The presence of 15 different genes encoding for virulence factors of *P. multocida* was investigated starting from different studies focused on pathogenicity [26,27,39,40]. Despite the possible compatibility between the annealing temperatures of the different primers, three Multiplex-PCR protocols (named A, B, and C) were staged because of the similarity of the molecular weight of the different amplicons. Details about the primer set used in the different Multiplex-PCRs are reported in Table 2.

The PCR mixture for Multiplex-PCRs consisted of 12.5 µL of 1X Platinum Mastermix (Thermo Scientific, Milan, Italy), 0.5 µL of each primer pair in a 50 pmol/µL primary concentration, and ultra-pure nuclease-free water (Thermo Scientific, Milan, Italy) until a final volume of 25 µL.

The thermal conditions previously reported in *Pasteurella multocida* identification paragraph (2.1.) were used for A, B, and C Multiplex-PCRs. All PCR products were visualized by agarose gel electrophoresis (1.5%) which was colored with ethidium bromide (0.5 μg/mL), using TBE (Tris-Borato-EDTA) as conductor. PCR results were read with Gel Doc-It (UVP) image analyzer.

### 2.5. Antibiotic Susceptibility Testing

The susceptibility of the identified bacterium to the antibiotics more frequently used in veterinary and human medicine to treat respiratory diseases and wounds from animals was evaluated. Therefore, the susceptibility to ciprofloxacin (CIP 5 μg/mL), sulfamethoxazole-trimethoprim (SXT 25 μg/mL), enrofloxacin (ENR 5 μg/mL), ampicillin (AMP 10 μg/mL), tetracycline (TE 30 μg/mL), nalidixic acid (NA 30 μg/mL), gentamicin (CN 10 μg/mL), amoxicillin-clavulanic acid (AMC 30 μg/mL), imipenem (IPM 10 μg/mL), cefotaxime (CTX 30 μg/mL), tilmicosin (TIL 15 μg/mL), amoxicillin (AX 25 μg/mL), doxycycline (DO 30 μg/mL), meropenem (MEM 10 μg/mL), azithromycin (AZM 15 μg/mL), and erythromycin (E 15 μg/mL) was determined. The standard Kirby–Bauer disk diffusion method on Muller–Hinton agar supplemented with 5% horse blood in aerobic and microaerophilic conditions was used according to European Committee on Antimicrobial Susceptibility Testing [41] and Clinical and Laboratory Standards Institute [42] standards. CLSI and EUCAST breakpoints were used to determine the susceptibility of the bacterium to the tested drugs.

## 3. Results

### 3.1. Pasteurella multocida Detection, Capsular Type, and Pathogenicity Pattern Identification

**Figure 4:**
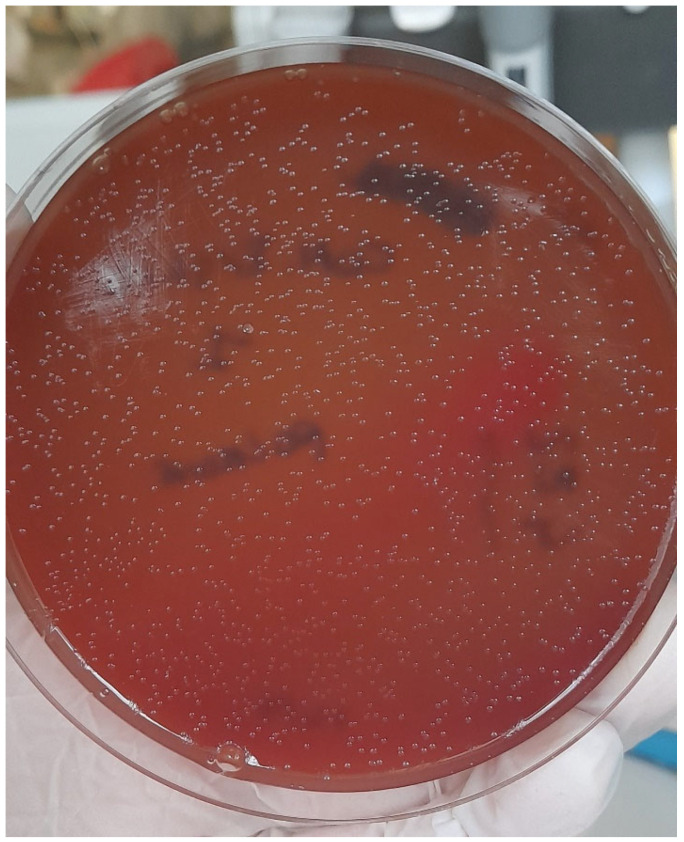
Microbiological findings from conjunctival sample: colonies were grown on blood agar and were identified as *Pasteurella multocida*.

The colonies appeared morphologically compatible with *Pasteurella* spp. (gray, translucent, and no-haemolytic) (Figure 4). No other kind of colony was found.

**Figure 5:**
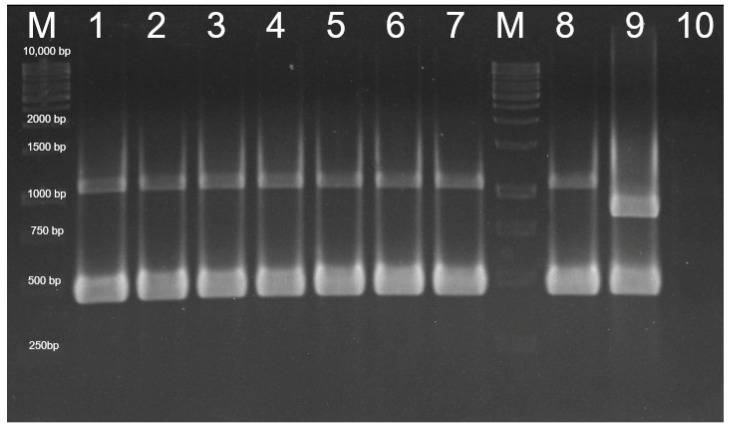
Identification of *P. multocida* type A by PCR: M: Marker (O’ Gene Ruler 1kb DNA Ladder, Ready to use, 250–10,000 bp, Thermo Scientific Inc.). Lanes 1, 2, 3, 4: conjunctival samples; Lanes 5, 6, 7: nasal samples; M: Marker; Lane 8: *P. multocida* type A positive control; Lane 9: *P. multocida* type F positive control; and Lane 10: negative control.

All tested colonies were identified as *P. multocida* belonging to capsular type A (Figure 5).

**Figure 6:**
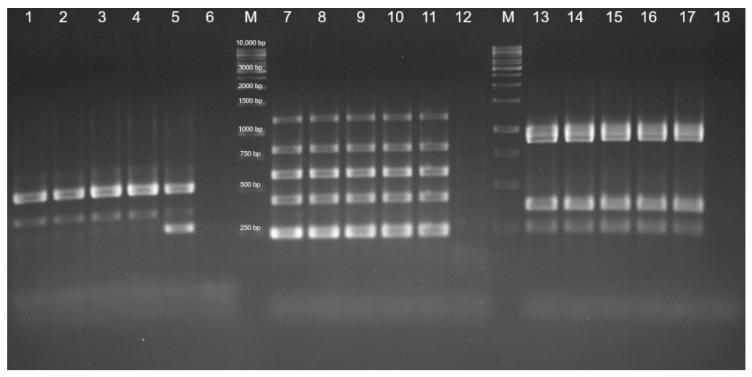
Detection of virulence genes of *P. multocida* by PCR. Protocol A: detection of SodA (361 bp), Fim4 (488 bp): Lanes 1, 2: conjunctival samples; Lanes 3, 4: nasal samples; Lane 5: positive control; Lane 6: negative control; M: Marker: O’ Gene Ruler 1kb DNA Ladder, Ready to use, 250–10,000 bp, Thermo Scientific Inc. Protocol B: detection of SodC (235 bp), TadD (416 bp), nanB1 (544 bp), HgbB (788 bp), ExbB (1144 bp): Lanes 7, 8: conjunctival samples; Lanes 9, 10: nasal samples; Lane 11: positive control; Lane 12: negative control; M: Marker. Protocol C: detection of Fur (244 bp), NanH1 (360 bp), FimA (866 bp), Oma87 (948 bp): Lanes 13, 14: conjunctival samples; Lanes 15, 16: nasal samples; Lane 17: positive control; and Lane 18: negative control.

Each strain tested positive for 11 different virulence genes: *fim4*, *fimA*, and *tadD*, responsible for fimbriae production and adhesion processes to host cells; *sodA*, *sodC*, *nanB1*, and *nanH1*, responsible for enzyme production, *hgbB*, *exbB*, *oma87*, and *fur*, involved in iron acquisition processes (Figure 6).

### 3.2. Antibiotic Susceptibility Testing

*Pasteurella multocida* was susceptible in vitro to all the antibiotics tested both in aerobic and microaerophilic conditions. Details on the results of antimicrobial susceptibility tests are reported in Table 3.

### 3.3. Outcome

The disease observed in the flock of pet rabbits was caused by a capsular type A *Pasteurella multocida* strain provided with a consistent set of genes associated with virulence. Based on the results of tests of susceptibility to antibiotics, the involved strain was susceptible to all tested antibiotics. Enrofloxacin was chosen for treatment because it is one of the most effective drugs to control the clinical forms of *Pasteurella multocida* in rabbits and is also authorized for use in pets.

Given the high number of rabbits to be treated, enrofloxacin was administered by drinking water (200 mg/L) for 15 days, obtaining the remission of rhinitis and conjunctivitis in bunnies and the improvement of symptoms in adults. One month after the end of the therapy, an inactive commercial vaccine was used to immunize the rabbits, obtaining a complete remission of the clinical form in the following six months.

## 4. Discussion

The correct diagnosis in veterinary medicine is of relevance for both animal and human health, considering that some pathogens can be transmitted to humans. *Pasteurella multocida* was identified as responsible for the syndrome observed in a flock of pet rabbits. *P. multocida* can be spread by affected animals mainly through nasal discharge. The effectiveness of the therapy is crucial to reducing the clinical symptoms and decreasing the spread of the bacteria involved in the disease process.

In vitro, the identified strain was susceptible to the antibiotics more frequently used in veterinary and human medicine, but the applied therapy appeared only partially efficacious in rabbits. Although enrofloxacin is the most suitable drug for treatments against *P. multocida* infections in rabbits, its in vivo efficacy is known to be lower than its in vitro efficacy. Therefore, the antibiotic treatment led only to the improvement of clinical conditions of the most heavily affected or chronically infected rabbits. Additionally, enrofloxacin was administrated by drinking water due to the high number of animals to be treated. Drinking water treatment can lead to non-uniform dosages because of different water intake by sick animals. In addition, after the antibiotic treatment, clinically healed rabbits often become carriers of *P. multocida,* with the possibility that the clinical signs will reappear later when adverse conditions occur [43].

These events could lead to possible risks for owners that may acquire *Pasteurella* infection from injuries caused by pets, but also by direct exposure to the bacteria [5,9,31,32]. Currently, rabbits are largely reared as pets and usually live in close contact with their owners. Nevertheless, these animals are timid, and scratches can frequently occur because they are often picked up and pampered. In addition, rabbits are often territorial, and may sometimes bite their owner, particularly when he/she manipulates the feeder or other cage equipment. Although *P. canis* and *P. dagmatis* may be found in bites from dogs and cats, *P. multocida* is the most prevalent species associated with human infections due to bites from companion animals [44].

*P. multocida* capsular type A was identified in the affected rabbits in this study. Type A is the serogroup prevalently associated with pasteurellosis in rabbits [25] and, interestingly, it is almost the only one detected in strains from humans [44,45], even type F, also found to be responsible for infections in rabbits [46], has been sporadically identified in humans [44,45]. Rabbits are very susceptible to *P. multocida* infection, which seems to affect them independent of gender and age. The clinical presentation of pasteurellosis in rabbits includes rhinitis, sinusitis, conjunctivitis, dacryocystitis, and pleuropneumonia, as well as otitis, encephalitis, and abscesses of the subcutaneous tissues or internal organs, bones, joints, and genitalia [47,48]. Symptoms frequently develop when immunosuppression, stress, or adverse environmental conditions occur. Nevertheless, strains equipped with several genes encoding for different virulence mechanisms usually have greater survival and invasiveness capabilities in the host [49,50,51] and are more frequently prone to induce clinical forms [28].

Many virulence genes involved in the pathogenicity of *P. multocida* such as fimbriae, adhesion and colonization factors (*ptfa*, *fimA*, *pfhA,* and *tadD*), iron regulation factors and protein acquisition (*exbB*, *exbD*, *tonB*, *hgbA*, *hgbB*, *tbpA*, and *fur*), superoxide dismutase (*sodA* and *sodC*), dermonecrotic toxins (*toxA*), a variety of Outer Membrane Proteins (*OMPs*) as protective factors (*ompA*, *ompH*, *omp87*, and *plpB*), and neuraminidase (*nanB* and *nanH*) have been proposed as possible markers of virulence [25,26,27,28,29]. According to studies carried out on *P. multocida* strains identified as responsible for disease in humans and other species [25,44], the strain isolated from the affected rabbits tested positive for several different virulence genes: *fim4*, *fimA*, and *tadD*, responsible for fimbriae production and adhesion processes to host cells; *sodA*, *sodC*, *nanB1*, and *nanH1*, responsible for enzyme production, and *hgbB*, *exbB*, *oma87*, and *fur*, involved in iron acquisition processes. This finding seems to explain the evidence of symptoms observed in the affected rabbits as well as some difficulties related to achieving the complete resolution of the syndrome that was obtained in the rabbit flock about 6 months after using the vaccine. The use of vaccines to immunize the rabbits against *P. multocida*, in particular, is recommended to minimize the risk of outcomes of the clinical form, but also to reduce the spread of bacteria by the infected animals. Therefore, rabbits should be routinely vaccinated against *P. multocida* when considering that they are reared as household pets and the transmission of the pathogen to humans cannot be excluded. Nevertheless, the vaccine is not able to prevent the infection, and bunnies that are sold to individuals and pet shops may appear healthy, even when they carry the bacterium. Therefore, screening for *P. multocida* should be considered as a part of the routine medical check of rabbits, particularly when they are purchased as companion animals for children and elder people. Additionally, typing the detected strains of the bacteria is important to assess their pathogenic potential and, consequently, to increase hygienic measures in the management of pet rabbits.

## 5. Conclusions

This study provides more knowledge about *Pasteurella multocida* detected in pet rabbits. We suggest the routine use of vaccines in pet rabbits and screening for *P. multocida* in the routine veterinary examination of rabbits, particularly when they will be used for pet therapy or kept as companion animals for children and elder people, considering that the transmission of the pathogen to humans cannot be excluded.

## Figures and Tables

**Table 1:** Identification of *Pasteurella multocida* species and capsular type by Multiplex-PCR: Primers and corresponding oligonucleotides sequences.

Target	Primers	Sequences	Amplimer Size
*Pasteurella multocida* specie	KMT1T7	ATCCGCTATTTACCCAGTGG	460 bp
	KMT1SP6	GCTGTAAACGAACTCGCCAC	
Capsular type A	CAPA-FWD	TGCCAAAATCGCAGTCAG	1044 bp
	CAPA-REV	TTGCCATCATTGTCAGTG	
Capsular type B	CAPB-FWD	CATTTATCCAAGCTCCACC	760 bp
	CAPB-REV	GCCCGAGAGTTTCAATCC	
Capsular type D	CAPD-FWD	TTACAAAAGAAAGACTAGGAGCCC	657 bp
	CAPD-REV	CATCTACCCACTCAACCATATCAG	
Capsular type E	CAPE-FWD	TCCGCAGAAAATTATTGACTC	511 bp
	CAPE-REV	GCTTGCTGCTTGATTTTGTC	
Capsular type F	CAPF-FWD	AATCGGAGAACGCAGAAATCAG	851 bp
	CAPF-REV	TTCCGCCGTCAATTACTCTG	

**Table 2:** A, B, and C protocols of Multiplex-PCR: investigated virulence genes, corresponding primers and nucleotide sequences, and molecular weights of amplicons.

	Virulence Factor	Primer	Sequence	Amplimer Size
A	Dermonecrotic toxin	ToxA-F ToxA-R	CTTAGATGAGCGACAAGGTT GGAATGCCACACCTCTATA	865 bp
	Trasferrin binding protein	TbpA-F TbpA-R	TTGGTTGGAAACGGTAAAGC TAACGTGTACGGAAAAGCCC	728 bp
	Type 4 fimbriae	Fim4-F Fim4-R	TGTGGAATTCAGCATTTTAGTGTGTC TCATGAATTCTTATGCGCAAAATCCTGCTGG	866 bp
	Superoxide dismutate	SodA-F SodA-R	TACCAGAATTAGGCTACGC GAAACGGGTTGCTGCCGCT	361 bp
	Filamentous hemagglutinin	Pfha-F Pfha-R	AGCTGATCAAGTGGTGAAC TGGTACATTGGTGAATGCTG	275 bp
B	Iron acquisition	ExbB-F ExbB-R	TTGGCTTGTGATTGAACGC TGCAGGAATGGCGACTAAA	283 bp
	Hemoglobin binding protein	HgbB-F HgbB-R	ACCGCGTTGGAATTATGATTG CATTGAGTACGGCTTGACAT	788 bp
	Neuroaminidase	NanB-F NanB-R	GTCCTATAAAGTGACGCCGA ACAGCAAAGGAAGACTGTCC	554 bp
	Putative nonspecific tight adherence protein D	TadD-F TadD-R	TCTACCCATTCTCAGCAAGGC ATCATTTCGGGCATTCACC	416 bp
	Lipoprotein B	PlpB-F PlpB-R	TTTGGTGGTGCGTATGTCTTCT AGTCACTTTAGATTGTGCGTAG	282 bp
	Superoxide dismutate	SodC-F SodC-R	AGTTAGTAGCGGGGTTGGCA TGTGCTGGGTGATCATCATG	235 bp
C	Outer membrane protein 87	Oma87-F Oma87-R	ATGAAAAAACTTTTAATTGCGAGC TGACTTGCGCAGTTGCATAAC	948 bp
	Fimbriae	FimA-F FimA-R	CCATCGGATCTAAACGACCTA AGTATTAGTTCCTGCGGGTG	866 bp
	Neuroaminidase	NanH-F NanH-R	GAATATTTGGGCGGCAACA TTCTCGCCCTGTCATCACT	360 bp
	ferric uptake regulation protein	Fur-R Fur-F	GTTTACCGTGTATTAGACCA CATTACTACATTTGCCATAC	244 bp

**Table 3:** Antibiotic susceptibility test performed on *P. multocida* detected in pet rabbit breeding.

		Diameter of Inhibition Zone (mm)	
Antibiotic (Acronym)	Susceptibility Breakpoint	Aerobic Conditions	Microaerophilic Conditions
Ciprofloxacin (CIP)	27	58	62
Trimetophim-Sulfametoxazole (SXT)	24	28	28
Enrofloxacin (ENR)	28	48	52
Ampicillin (AMP)	27	32	30
Tetracycline (TE)	24	46	44
Nalidixic acid (NA)	23	50	52
Gentamicin (CN)	20	22	26
Amoxicillin-Clavulanic acid (AMC)	27	36	30
Imipenem (IPM)	23 *	S	40
Cefotaxime (CTX)	26	S	54
Tilmicosin (TIL)	14	28	27
Amoxicillin (AX)	17	33	36
Doxycycline (DO)	23	36	40
Meropemem (MEM)	23 *	42	48
Azitromycin (AZM)	20	44	40
Erytrhomycin (E)	27	32	36

* Susceptibility breakpoint referred to *Enterobacteriaceae* due to lacking specific values for *P. multocida.*

## Data Availability

The data presented in this study are available on request from the corresponding author.
